# HOXC8: a predictive glioma biomarker that induces epithelia-mesenchymal transition

**DOI:** 10.1186/s41016-018-0132-9

**Published:** 2018-08-28

**Authors:** Tingyu Liang, Xiaoxuan Wang, Peiliang Li, Yang Cao, Enshan Feng, Gan You

**Affiliations:** 1 Department of Neurosurgery, Beijing Tiantan Hospital, Capital Medical University, No. 6 TiantanXili, Dongcheng District, Beijing, 100050 China; 2 Department of Neurosurgery, Beijing Ditan Hospital, Capital Medical University, Beijing, 100020 China; 3 Capital Medical University, Beijing, 100050 China; 4 Department of Neuropathology, Beijing Neurosurgical Institute, Capital Medical University, No. 6 TiantanXili, Dongcheng District, Beijing, 100050 China

**Keywords:** HOXC8, Cell cycle, Migration, Invasion, EMT

## Abstract

**Background:**

The transcription factor Homeobox C8 (*HOXC8*) is overexpressed and regulates many important genes involved in the proliferation and invasion of many malignant tumors. However, the function of HOXC8 in gliomas remains unclear.

**Method:**

Based on the Chinese Glioma Genome Atlas (*CGGA*) set, HOXC8 expression is negatively correlated with overall survival (*OS*). Small interfering RNA (*si-HOXC8*) was used to downregulate the mRNA and protein expression levels of HOXC8 to assess glioma cell proliferation, migration and invasion.

**Results:**

Patients with higher HOXC8 levels showed poorer prognosis. DAVID analysis results indicated that HOXC8 was related to cell cycle, cell adhesion and immune response. In U251 and LN229 glioma cells treated with small interfering RNA for HOXC8 (*si-HOXC8*) for gene knockdown, significantly lower cell capacity of growth, migration and invasion was observed. Moreover, HOXC8 knockdown could reduce the protein expression of classical epithelial mesenchymal transition (*EMT*) related markers.

**Conclusion:**

HOXC8 may play an important role in glioma proliferation, migration and invasion. These findings indicated that HOXC8 may constitute a novel target for glioma treatment.

## Background

Gliomas account for most of malignant tumors of the central nervous system (*CNS*). Despite the recent progress in surgical resection, radiotherapy and chemotherapy, prognosis for glioma remains poor [1, 2]. Gliomas possessed strong proliferation, migration and invasion abilities, causing high mortality. Therefore, multiple studies have identified key genes that could help achieve a breakthrough in gliomas treatment [3, 4]. In this study, Homeobox C8 (*HOXC8*), a transcript factor, was selected for in-depth assessment.

HOXC8, located in a cluster on chromosome 12, belongs to the 39-member HOX family of proteins [5]. Under normal circumstances, HOXC8 plays an indispensable role in embryonic morphogenesis and system morphogenesis [6, 7]. However, in various tumors, including ovarian cancer, hepatocellular carcinoma, and breast cancer, HOXC8 induces cell proliferation, migration and invasion, and is inversely correlated with overall survival (*OS*) through different mechanisms [8-10]. Although HOXC8 is important in tumorigenesis, its mechanism in glioma remains unclear.

Epithelial mesenchymal transition (*EMT*), a cellular alteration that confers a more invasive and drug-resistant phenotype, was initially observed in embryonic development [11, 12]. EMT was a complex process reflected by epithelial cells acquiring the mesenchymal phenotype and motility through a cascade of biological events [13]. In gliomas, the mechanism by which HOXC8 affected EMT is unclear.

In summary, the aim of our study was to assess the oncogenic function of HOXC8. HOXC8 knockdown could inhibit glioma cell proliferation, migration and invasion. Therefore, HOXC8 could provide novel insights for glioma treatment.

## Methods

### Dataset and glioma samples

We obtained mRNA samples and follow-up information from the Chinese Glioma Genome Atlas (*CGGA*). OS was defined as the time from surgery to death. Glioma samples were collected from surgical resection, snap-frozen in liquid nitrogen and stored at − 80 °C until RNA extraction [14]. This study was approved by the Ethics Committee of Beijing Tiantan Hospital, and written informed consent was obtained from all the patients.

### Cell culture and siRNA transfection

Two glioma cell lines were cultured in Dulbecco’s Modified Eagle’s Medium (*DMEM*) supplemented with 10% fetal bovine serum (*FBS*) and antibiotics (*100 U/ml penicillin and 100* μg*/mL streptomycin*), in a humidified atmosphere with 5% CO2 at 37 °C [15]. The siRNA segmentand a non-specific control siRNA sequence (*NC*) were purchased from Genepharma (*Shanghai, China*), and transfected into LN229 and U251 cells using Lipofectamine™ 2000 (*Invitrogen, Shanghai, China*), according to the manufacturer’s instructions. After 48 h of incubation, samples were collected for RT-PCR and Western blot assay [16].

### Real-time polymerase chain reaction (*RT-PCR*) and western blot (*WB*)

RT-PCR and Western blot were performed as previously described [4, 17].The following primers were used for RT-PCR: HOXC8, Forward 5’-ACCGGCCTATTACGACTGC-3′ and Reverse 5′-TGCTGGTAGCCTGAGTTGGA-3′; GAPDH (Forward 5′- GGAGCGAGATCCCTCCAAAAT -3′ and Reverse 5′- GGCTGTTGTCATACTTCTCATGG-3′ was used as an internal control, and fold changes were calculated by the 2 − ΔΔCt method. For Western blotting, densities of specific protein bands were quantified after normalization to Tubulin or Actin level in the same sample.

### Clone formation assay

A total of 1000 cells were seeded per well in a 6-well plate in DMEM and 10% FBS, and cultured for 2 weeks before Crystal violet stain. Then, clone numbers were recorded in siRNA and control groups, respectively.

### Transwell migration assay

Transwell plates were used to study cell migration and invasion. A total of 1 × 10^5^ LN229 or U251 cells were seeded in each well in DMEM only. Control medium was added to the lower chamber of the transwell plates. After 36 h (*invasion assay*) or 12 h (*migration assay*) of incubation, the cells in upper chambers were removed carefully before Crystal violet staining. Analysis was carried out by microscopy, counting cells in siRNA and control groups, respectively [18].

### Statistical analysis

We used Kaplan-Meier analysis (*log-rank test*) to evaluate predictive value of HOXC8 expression for OS in different groups. Univariate and multivariate Cox regression analyses were used to determine that HOXC8 was an independent factor to predict OS. Pearson relation analysis was performed in CGGA array set using R language package. All values were considered statistically significant at *p* < 0.05.

## Results

### HOXC8 was associated with overall survival in gliomas

**Fig. 1:**
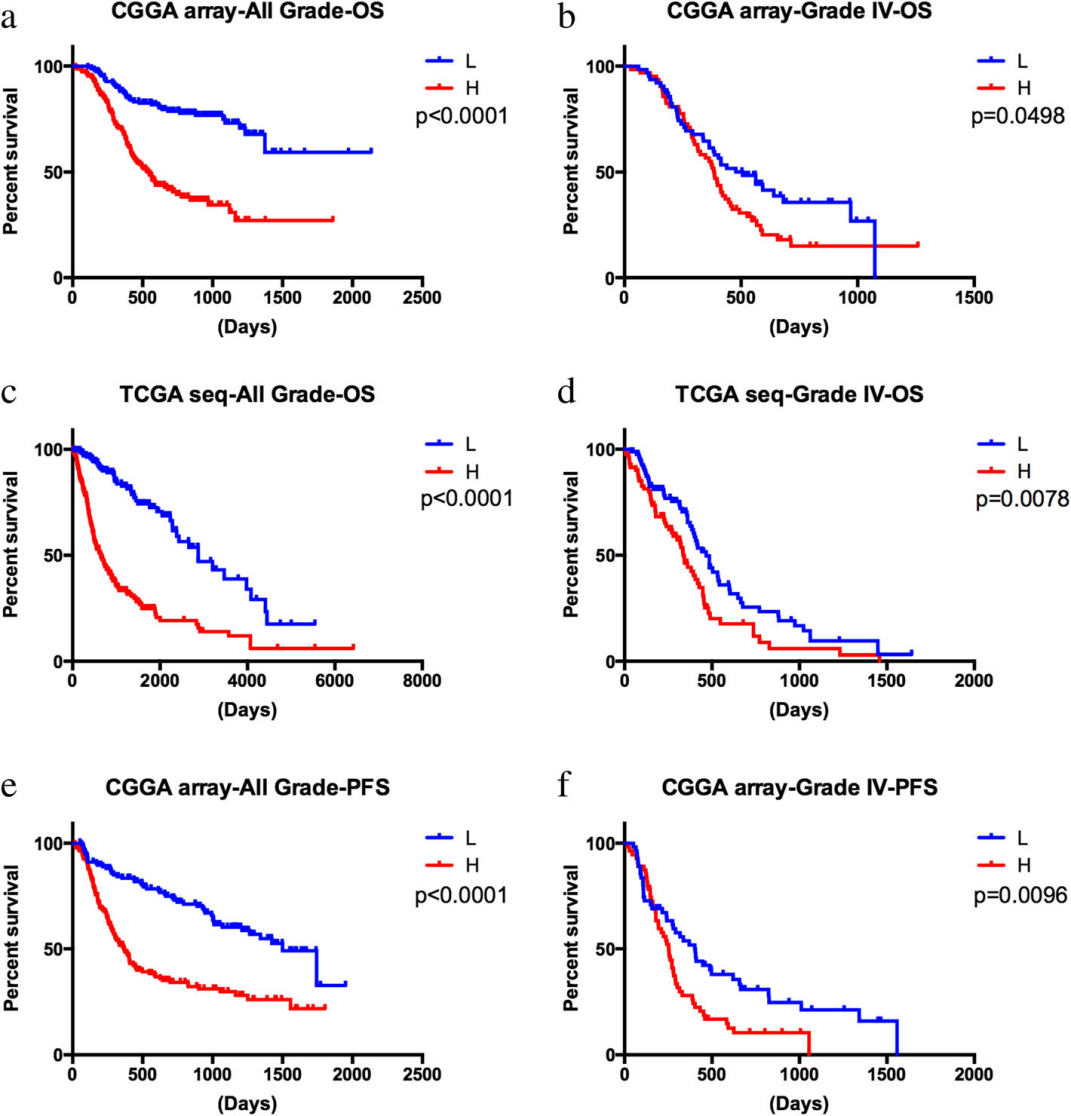
**a**-**d** HOXC8 expression was negatively related with OS in all grade gliomas and grade IV gliomas repectively in CGGA and TCGA sets (*p < 0.05*); **e**-**f** HOXC8 expression was negatively related with PFS in all grade gliomas and grade IV gliomas repectively in CGGA set (*p < 0.05*); OS: overall survival; PFS: progression-free survival

We obtained the HOXC8 mRNA expression and prognosis information from the CGGA mRNA array and TCGA RNA-seq datasets, and assessed the association of HOXC8 expression with overall survival (*OS*). As shown in Fig. 1a and b, the level of HOXC8 mRNA expression was negatively related with OS in all grade gliomas and grade IV gliomas (*p < 0.05*). Moreover, we further validated the results in TCGA RNA-seq set (Fig. 1c and d*, p < 0.05*). Moreover, in Fig. 1e and f, in CGGA array set, progression-free survival time of was shorter in higher HOXC8 expression group *(p < 0.05).* To further validation our results, we performed a univariate Cox regression analysis, the results showed that HOXC8 was showed a risk factor for glioma patients (*p < 0.0001, HR = 1.545,* Table 1). What is more, age at diagnosis, IDH1 status, gliomas grade, TCGA subtype was significantly related with survival time of glioma patients (*p < 0.0001,* Table 1). Then, the results from multivariate Cox regression analysis including HOXC8 expression, age at diagnosis, IDH1 status, gliomas grade and TCGA subtype showed that HOXC8 was an independent prognostic factor for glioma patients (*p < 0.0001, HR = 1.247,* Table 1).

**Table 1:** Univariate and multivariate Cox analysis in CGGA glioma samples

Clinical factors	Univariate			P	Multivariate			P
	HR	95%CI			HR	95%CI		
		Lower	Upper			Lower	Upper	
Age at diagnosis	1.044	1.026	1.062	< 0.0001	1.011	0.993	1.029	0.240
Gender (male)	1.162	0.792	1.704	0.443				
IDH1 status (mutation)	0.335	0.219	0.511	< 0.0001	0.932	0.544	1.597	0.799
High HOXC8 expression	1.545	1.397	1.709	< 0.0001	1.247	1.110	1.402	< 0.0001
Grade (high grade)	3.960	2.960	5.299	< 0.0001	3.176	2.307	4.371	< 0.0001
TCGA subtype (mesenchymal type)	1.605	1.362	1.890	< 0.0001	1.048	0.866	1.267	0.631

IDH1: isocitrate dehydrogenase 1; CGGA: Chinese Glioma Genome Atlas; TCGA: The Cancer Genome Atlas; CI: Confidence Interval

In conclusion, we hypothesized that HOXC8 could serve as a novel biomarker for predicting glioma OS.

### Gene functional analysis

**Fig. 2:**
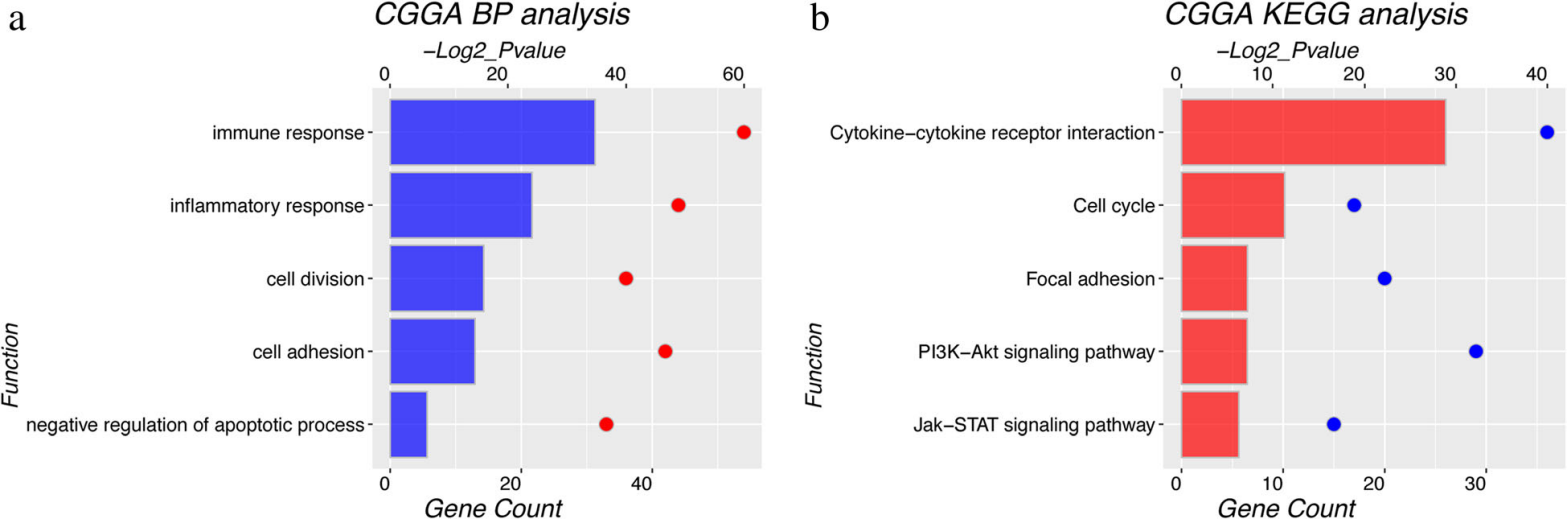
HOXC8 associated genes and their potential function **a** GO analysis; **b** KEGG pathway analysis. GO: Gene Ontology

The function of HOXC8 in glioma remains unclear. We performed Pearson relation analysis in CGGA array set using R language package. To explore differential biological function of HOXC8, we selected top 1000 genes positively or negatively related with HOXC8 for DAVID analysis respectively. In Fig. 2a and b, Gene Ontology (*GO*) and KEGG pathways analysis showed that cell cycle, cell adhesion, immune response and inflammatory response were high expression in high-HOXC8 expression group. Meanwhile, taken together, gliomas in high-HOXC8 group had a greater capacity for proliferation, migration and invasion than these in low-HOXC8 group.

### LN229 and U251 glioma cell lines were used to investigate biological processes

**Fig. 3:**
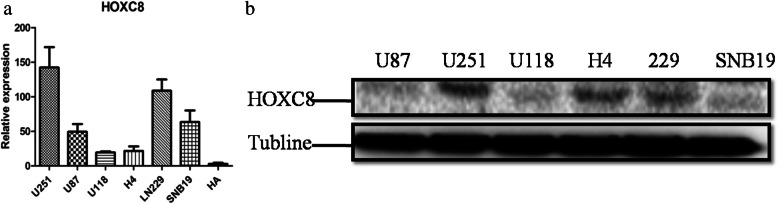
HOXC8 expression in different glioma cell lines. **a** mRNA expression level of HOXC8 in six glioma cell lines (*LN229, U87, U118, U251, SNB19, H4*) and one normal human astrocyte (*HA*); **b** Protein expression level of HOXC8 in six glioma cell lines

Then in Fig. 3a and b, seven (six glioma cell lines and one normal human astrocyte) frequently-studied cell lines (*LN229, U87, U118, U251, SNB19, H4, and HA*) were selected for HOXC8 mRNA and protein expression assessment. Interestingly, the six glioma cell lines showed higher HOXC8 expression levels compared with HA cells (*human astrocytes*). More importantly, LN229 and U251 cells had highest endogenous mRNA and protein levels of HOXC8. Based on the above results, LN229 and U251 were selected for subsequent experiments.

### HOXC8 could significantly promote glioma proliferation

**Fig. 4:**
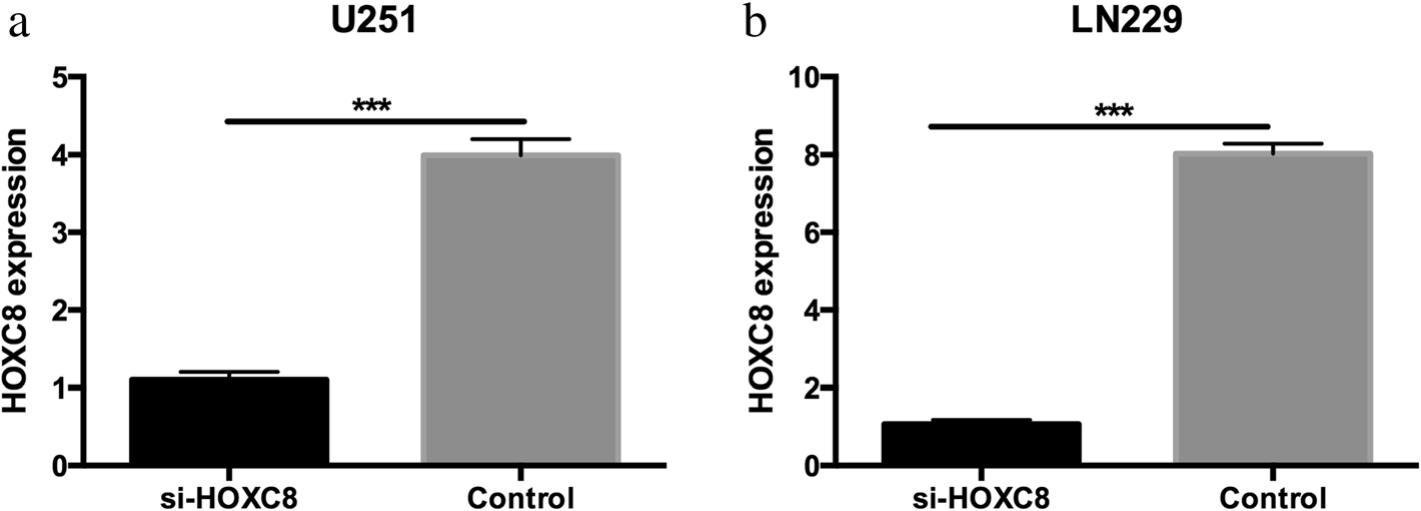
Small interfering RNA of HOXC8 (*si-HOXC8*) could significantly knocked down HOXC8 mRNA expression in U251 (**a**) and LN229 (**b**) glioma cell lines (*p < 0.05*)

**Fig. 5:**
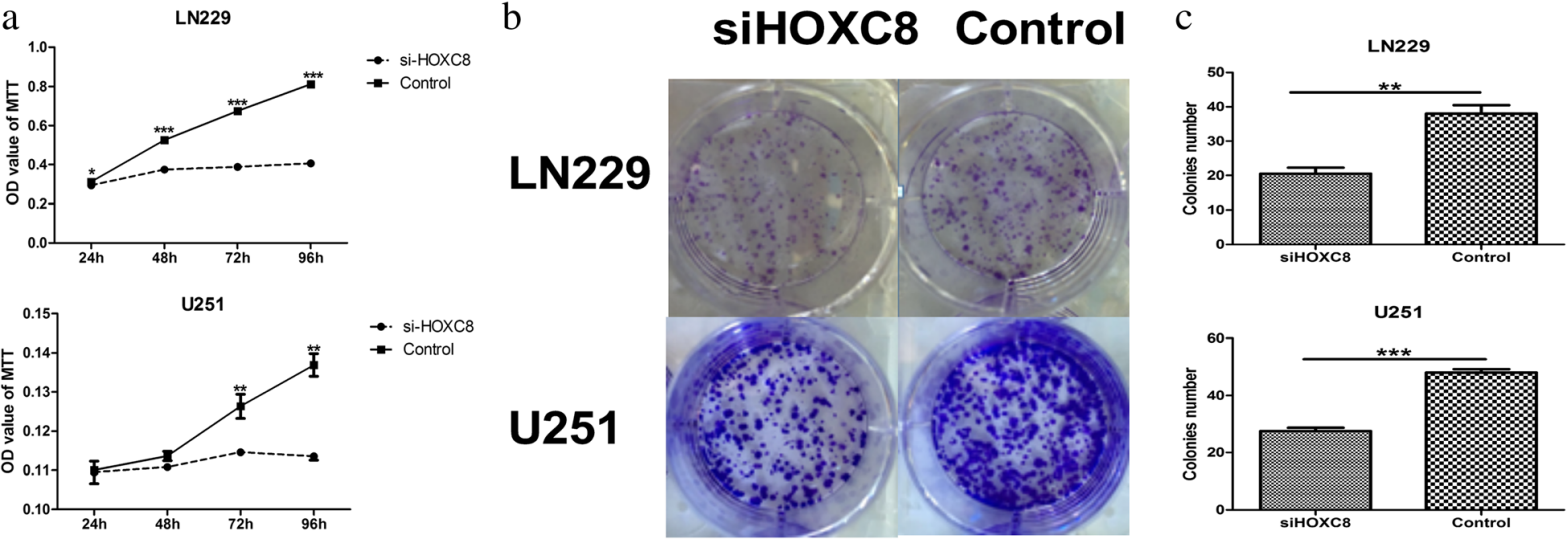
**a** MTT assay indicates that si-HOXC8 can suppress LN229 and U251 cell proliferation (*p < 0.05*); **b** & **c** Clonogenic assay indicates that si-HOXC8 can suppress LN229 and U251cell clone formation (*p < 0.05*)

To assess the effect of HOXC8 on cell proliferation, HOXC8 was silenced in LN229 and U251 cells by siRNA; 48 h after transfection, siRNA successfully knocked down HOXC8 at the mRNA levels (Fig. 4a and b). Cell viability was measured by MTT and clonogenic assays, as shown in Fig. 5a-c, antagonism of HOXC8 expression suppressed the cell growth and the formation of cell clones (*p < 0.05*).

### Downregulation of HOXC8 inhibits cell migration and invasion by blocking the EMT pathway

**Fig. 6:**
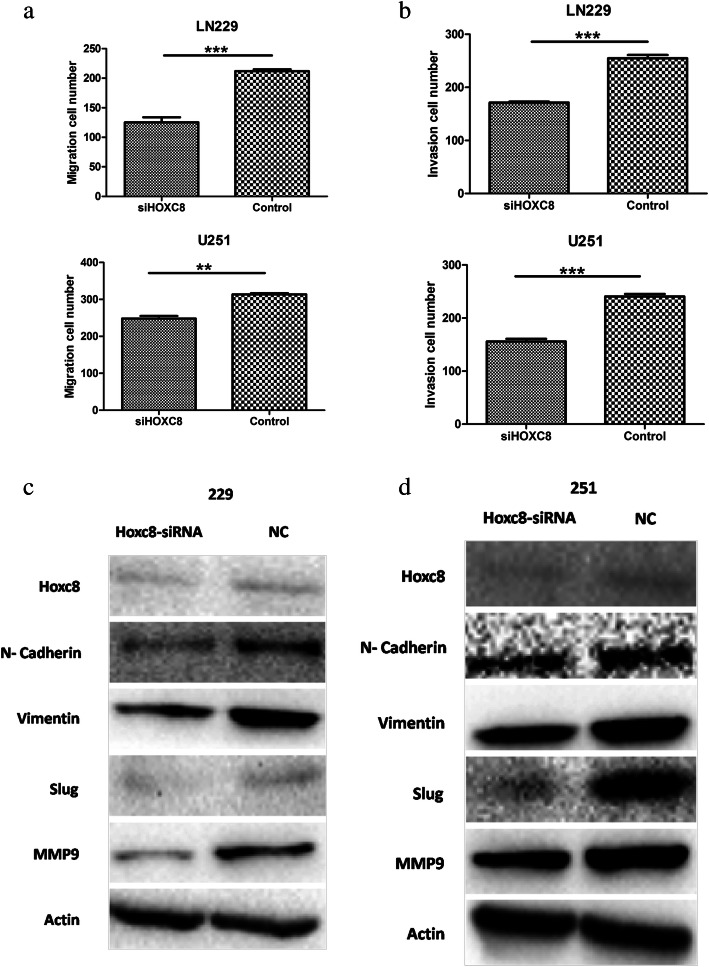
Si-HOXC8 inhibited glioma cell migration, invasion and EMT process **a** & **b** The number of migration and invasion LN229 and U251 cells were decreased in si-HOXC8 group (*p < 0.05*); **c** & **d** Si-HOXC8 could significantly decrease EMT related marker including N-cadherin, SLUG, VIMENTIN, MMP9. EMT: epithelial mesenchymal transition

To assess whether the migration and invasion capacity of glioma cells could be inhibited by si-HOXC8, we performed migration and Matrigel invasion assays using LN229 and U251 cells. Crystal violet staining showed markedly less migration and invasion glioma cells after HOXC8 silencing compared with negative-control values (*p < 0.01;* Fig. 6a and b). In addition, EMT was a novel mechanism involved in metastasis [19, 20]. The protein expression levels of mesenchymal markers (*N-Cadherin, Slug, Vimentin and MMP9*) were reduced in si-HOXC8 LN229 and U251 cells compared with the control group, as assessed by Western blot (Fig. 6c and d). Taken together, si-HOXC8 suppressed cell migration and invasion by reversing EMT.

## Discussion

Massive evidence supports that HOXC8 plays an important role in malignant tumor formation and progression [21, 22]. As a transcription factor, HOXC8 is able to regulate and coordinate multiplevital genes (e.g. *Mgl1, Embigin, Meis1, and Fyn*) involved in cancer development and progression [8, 22, 23]. Previous findings demonstrated that HOXC8 depletion by small interfering RNA suppresses epithelial ovarian cancer proliferation and migration, and induces apoptosis by increasing ZAC1 expression [24]. HOXC8, overexpressed in hepatocellular carcinoma (*HCC*) compared with adjacent non-tumor tissues, is associated with poor prognosis. Si-HOXC8 blocks G1-S phase transition, inhibits cell proliferation and renders cells more sensitive to oxaliplatin [9]. Therefore, HOXC8 as a potential oncogenic driver, plays an important role in cancer cell proliferation, migration and invasion, by overregulating many oncogenes and downregulating tumor suppressor genes. In this study, we demonstrated that si-HOXC8 blunted glioma cell proliferation, migration and invasion by reversing EMT.

EMT is known to be implicated in cancer progression, metastasis and drug resistance [25]. EMT reversibly enables polarized epithelial cells to lose their epithelial characteristics and to acquire mesenchymal properties [13]. The availability of antibodies targeting epithelial (*E-cadherin, β-catenin, and Claudin-1*) and mesenchymal (*N-cadherin, Slug, Vimentin and MMP9*) markers makes it convenient to assess EMT [26]. Multiple genes and pathways were reported to be involved in EMT [26-28]. However, the mechanisms by which HOXC8 affects EMT remains unclear. This study found that the HOXC8 was involved in EMT. Moreover, si-HOXC8 decreased the expression of classical mesenchymal markers (*N-cadherin, Slug, Vimentin and MMP9*). Hence, the present findings provide additional insights regarding the implication of HOXC8 and target genes in EMT as well as malignant tumor progression.

In summary, this study demonstrated that higher HOXC8 expression resulted in shorter OS. In agreement, HOXC8 knockdown inhibited glioma cell proliferation. Meanwhile, reduction of HOXC8 could also affected migration and invasion through blocking EMT pathway. The present findings suggested that HOXC8 should be considered a novel biomarker and target for glioma treatment.
